# Water deficit changes the relationships between epidemiological traits of Cauliflower mosaic virus across diverse *Arabidopsis thaliana* accessions

**DOI:** 10.1038/s41598-021-03462-x

**Published:** 2021-12-16

**Authors:** Sandy E. Bergès, Denis Vile, Michel Yvon, Diane Masclef, Myriam Dauzat, Manuella van Munster

**Affiliations:** 1 LEPSE, Univ Montpellier, INRAE, Institut Agro, Montpellier, France; 2 PHIM, Univ Montpellier, CIRAD, INRAE, Institut Agro, Montpellier, France

**Keywords:** Microbiology, Plant sciences

## Abstract

Changes in plant abiotic environments may alter plant virus epidemiological traits, but how such changes actually affect their quantitative relationships is poorly understood. Here, we investigated the effects of water deficit on Cauliflower mosaic virus (CaMV) traits (virulence, accumulation, and vectored-transmission rate) in 24 natural *Arabidopsis thaliana* accessions grown under strictly controlled environmental conditions. CaMV virulence increased significantly in response to water deficit during vegetative growth in all *A. thaliana* accessions, while viral transmission by aphids and within-host accumulation were significantly altered in only a few. Under well-watered conditions, CaMV accumulation was correlated positively with CaMV transmission by aphids, while under water deficit, this relationship was reversed. Hence, under water deficit, high CaMV accumulation did not predispose to increased horizontal transmission. No other significant relationship between viral traits could be detected. Across accessions, significant relationships between climate at collection sites and viral traits were detected but require further investigation. Interactions between epidemiological traits and their alteration under abiotic stresses must be accounted for when modelling plant virus epidemiology under scenarios of climate change.

## Introduction

As sessile organisms, plants are continuously exposed to combinations of abiotic and biotic stresses^1^. In response to these constraints, plants have evolved specific mechanisms to detect environmental changes and respond to complex stress conditions, minimizing damage while maintaining valuable resources for growth and reproduction^2,3^. Soil water deficit (WD) and virus infection are two important factors impacting plant performance that frequently occur simultaneously in natural conditions^4,5^.

Viruses are obligate parasites and, as such, cannot complete their life cycle without exploiting a suitable host. Hence, the success of viral infection depends on the physiological machinery of the host plant, and thus any environmental change that affects plant physiology—mostly through alteration of host signaling pathways—may also affect viral infection outcomes^6^. Thus, in the context of climate change, recent studies have focused on important viral life traits (e.g. accumulation, virulence, vectored-transmission rate) in the tripartite plant-vector-pathogen interaction field^7–10^.

Virulence, defined here as the degree of damage caused to a susceptible host plant, may be affected by environmental factors such as air temperature, edaphic water availability, and atmospheric CO_2_^10–12^. Specific environmental conditions can also modulate virulence in such a way that viral infection is positively correlated with host fitness^13–19^. We have also shown recently that virulence can increase under water deficit^20^.

The contrasting effect of abiotic stresses on viral accumulation through the hijacking of plant signaling and defense pathways has received much recent attention^11,21–23^. For instance, while Barley yellow dwarf virus titer increases by more than 30% in wheat grown under elevated atmospheric CO_2_ (eCO_2_) or elevated temperature^22,24^, titers of Soybean mosaic virus or Cauliflower mosaic virus (CaMV) are negatively impacted in plants grown under water deficit^10,25^. However, no clear trends were described between viral accumulation and virulence under these challenging conditions.

Most plant viruses rely on arthropods to spread, with aphids being by far the most widespread vectors^26^. Virus acquisition from an infected plant, virus retention, and virus inoculation to a new plant are critical steps that mediate the success of the infection^27^. Virus transmission efficiency depends not only on the intrinsic strategy of the virus and vector behavior, but also on the physiological status of the host plant, the environment, and thus on tripartite plant-vector-pathogen interactions^28–30^. For example, vectored-transmission of CaMV and Turnip mosaic virus increased significantly when *Brassica rapa* source plants experienced a severe water deficit^31^. The same abiotic stress applied to *Arabidopsis thaliana* (L.) Heynh (Col-0 accession) infected with Turnip yellows virus, drastically reduced transmission efficiency, likely due to an altered feeding behavior of the aphid vector^32^.

The viral traits discussed above are expected to interact tightly, allowing successful coevolution of hosts and pathogens, as originally described through the trade-off hypothesis^33,34^. This hypothesis predicts the existence of an optimal level of virulence that integrates the benefits of transmission and the cost of killing the host and, so for, relies on two key assumptions: (1) a positive correlation between accumulation and transmission, and (2) a positive correlation between accumulation and virulence^33,35^. While the first assumption has been demonstrated for several vector-borne plant viruses, the latter remains controversial^36–39^. Overall, available data do not allow a prevailing correlation between these three viral traits in plant viral pathosystems to be confirmed or refuted^35,40^. Moreover, data on how these different viral traits would interact in a disturbed environment are scarce.

In a previous study, we have shown that water deficit altered the relationship between transmission rate and virulence of CaMV in *A. thaliana*^10^. However, the restricted number of accessions assessed precluded analysis of other viral trait relationships that may help explain the transmission–virulence trade-off.

Here, we examined the effect of an edaphic water deficit on viral traits when *A. thaliana* plants were infected with CaMV. Relationships between virulence, as quantified by the relative change in aboveground dry mass, accumulation and aphid transmission, were analyzed under both well-watered and water deficit conditions. We quantified these viral traits in a set of 24 natural *A. thaliana* accessions originating from the Iberian Peninsula for the following reasons: (1) this region is characterized by a wide range of climatic conditions, notably in terms of annual precipitation, and (2) CaMV has been found to infect natural populations of *A. thaliana*^41^. These accessions were grown under strictly controlled environmental conditions in the high-throughput phenotyping platform PHENOPSIS^42^. We previously demonstrated a significant variation in CaMV virulence among these specific *A. thaliana* accessions that was not related to climatic variables, specifically annual precipitation, of original local populations^10,20^. Here, we explicitly tested the trade-off hypothesis, namely that virulence is positively related to vectored-transmission rate whatever the conditions of water availability and discuss the observed relationships between viral traits and climatic variables.

## Results

The CaMV isolate Cabb B-JI successfully infected plants from all 24 *A. thaliana* accessions grown in two independent experiments, regardless of watering treatment. Infected plants showed characteristic symptoms of a CaMV infection: chlorotic lesions, vein-clearing and crinkled rosette leaves.

### Changes in viral traits under water deficit

**Figure 1:**
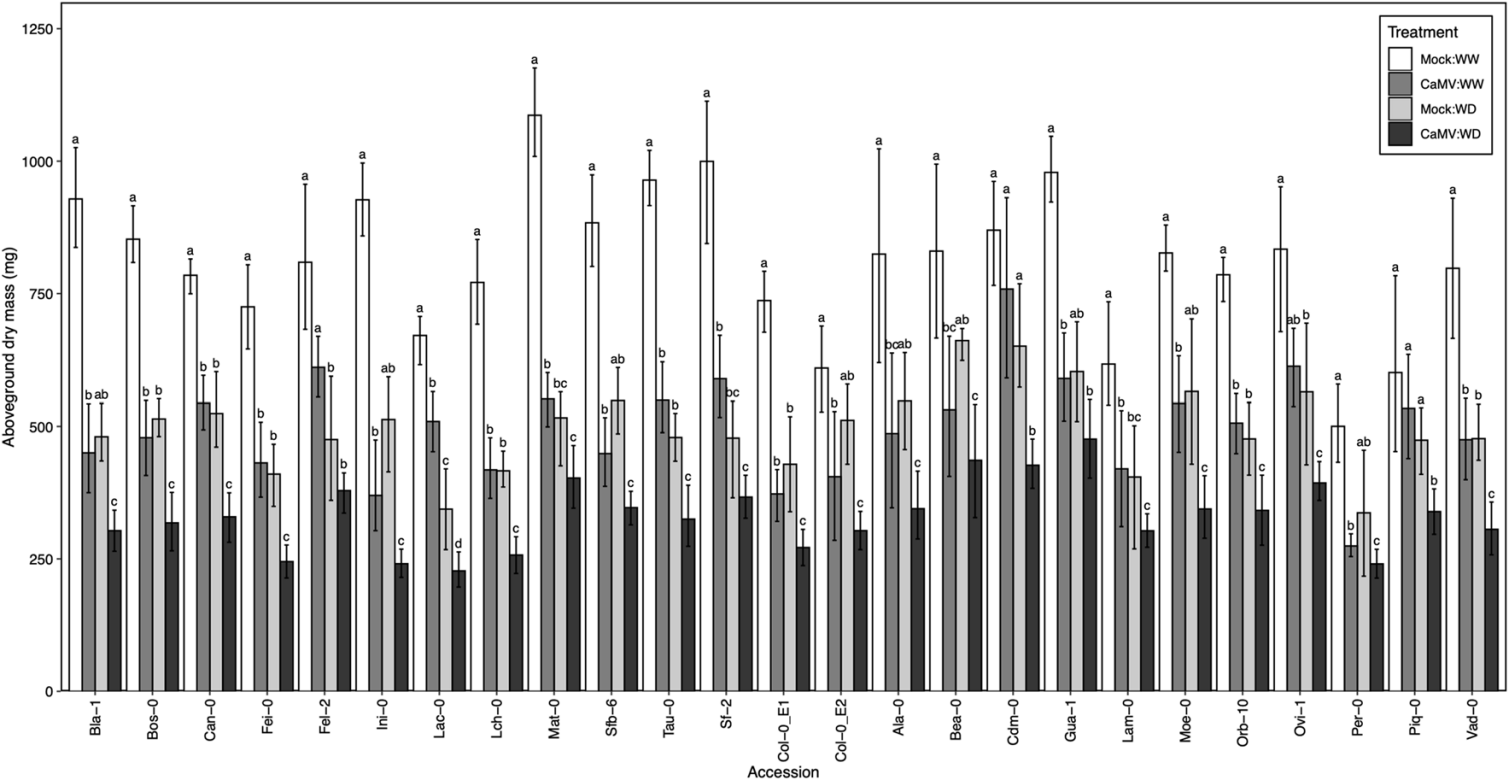
Effects of CaMV infection and watering treatment on vegetative growth of 24 *A. thaliana* accessions. Bars are mean ± 95% CI of rosette dry mass (mg) at 30 dpi of plants grown under well-watered (WW) and water deficit (WD) treatments: mock-inoculated:WW (white bars; *n* = 4), mock-inoculated:WD (dark grey bars; *n* = 4), CaMV-infected:WW (light grey bars; *n* = 15) and CaMV-infected:WD (black bars; *n* = 15) treatments. Data are from Experiment 1 and 2 (experiment 1, *n* = 13; experiment 2, *n* = 12 accessions). Different letters indicate a significant difference between treatments for each accession. Accessions are ordered alphabetically by experiment from Bla-1 to Sf-2 (experiment 1) and from Ala-0 to Vad-0 (experiment 2). Col_E1: Col-0 of experiment 1 and Col_E2: Col-0 of experiment 2.

**Figure 2:**
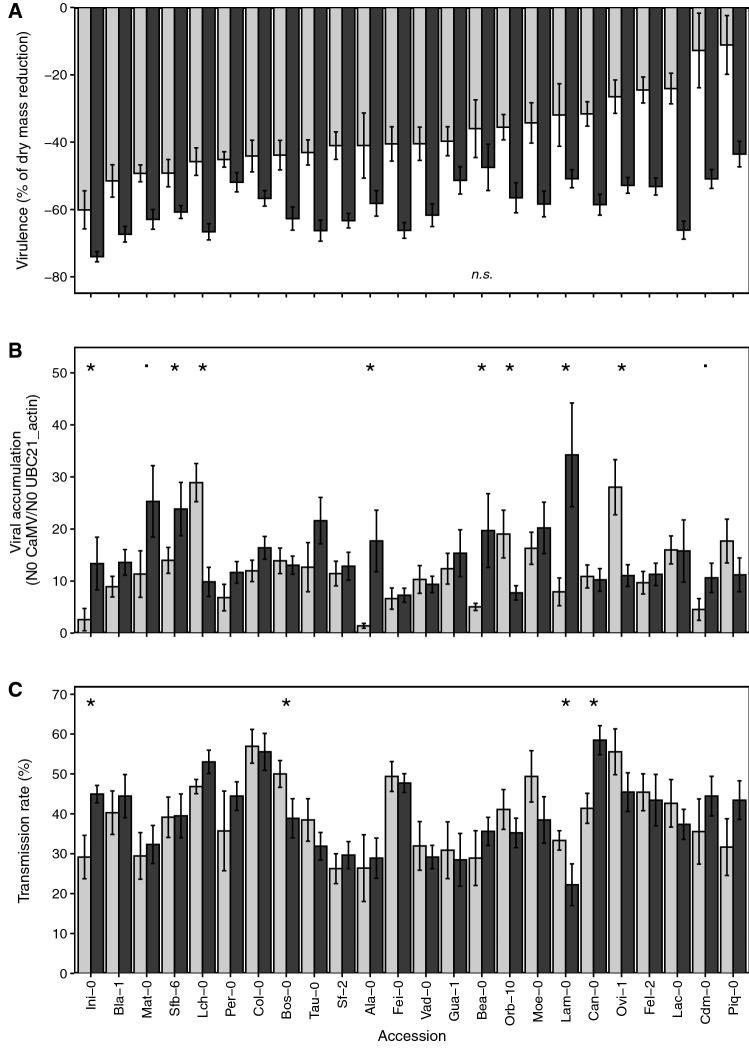
CaMV traits of *A. thaliana* accessions under well-watered (WW) and water deficit (WD) treatments. (A) CaMV virulence determined as vegetative growth reduction (%) (*n* = 15; per accession and per treatment). (B) CaMV-accumulation in source plants (*n* = 11 per accession and per treatment). (C) Transmission rate, i.e., mean proportion of infected receptor plants (*n* = 9 receptor plants per source plant, 11 source plants per accession and per treatment). Data are from experiment 1 and 2 (experiment 1, *n* = 13; experiment 2, *n* = 12 accessions). Accessions are ordered by virulence level under well-watered conditions. Bars and error bars are means ± se of plant grown under CaMV-infected:WW (light gray bars) and CaMV-infected:WD (black bars) treatments at 30 dpi. (A), all differences between watering treatments are significant but Bea-0 (*n.s.*: not significant). (B, C), statistically significant differences between watering treatments for each accession following Wilcoxon test are indicated (*: *P* < 0.05; : *P* < 0.10).

For each of the 24 accessions of the *A. thaliana* plant species, we determined vegetative growth, virulence (expressed as reduction of aboveground rosette dry mass), viral accumulation and transmission efficiency by aphids in CaMV-source plants under well-watered (WW) and water deficit (WD) treatments. Under well-watered conditions, mean aboveground dry mass of mock-inoculated (± se) ranged from 500 ± 40 mg (Per-0) to 1087 ± 50 mg (Mat-0) (Fig. 1, Supplementary Table S1). CaMV infection induced a reduction in vegetative growth in all *A. thaliana* accessions (Figs. 1, 2A; *P* < 0.001, Supplementary Table S2)*.* Moreover, a highly significant variation in accession responses to CaMV infection and WD was found (Figs. 1, 2), as indicated by a significant interactive effect between accession and inoculation, and between accession and watering (Supplementary Table S3; *P* = 0.013 and *P* = 0.021, respectively). Under the WW treatment, CaMV virulence varied greatly, with some accessions exhibiting less than 11.1 ± 8.8% reduction in vegetative growth (Piq-0), while in the most CaMV-susceptible accessions vegetative growth decreased up to 60.1 ± 5.7% (Ini-0) (–38% on average across accessions; Fig. 2A). As expected from Fig. 1, the combination of viral infection and WD was even more detrimental to vegetative growth of all accessions (–58% on average across accessions; Fig. 2A).

**Figure 3:**
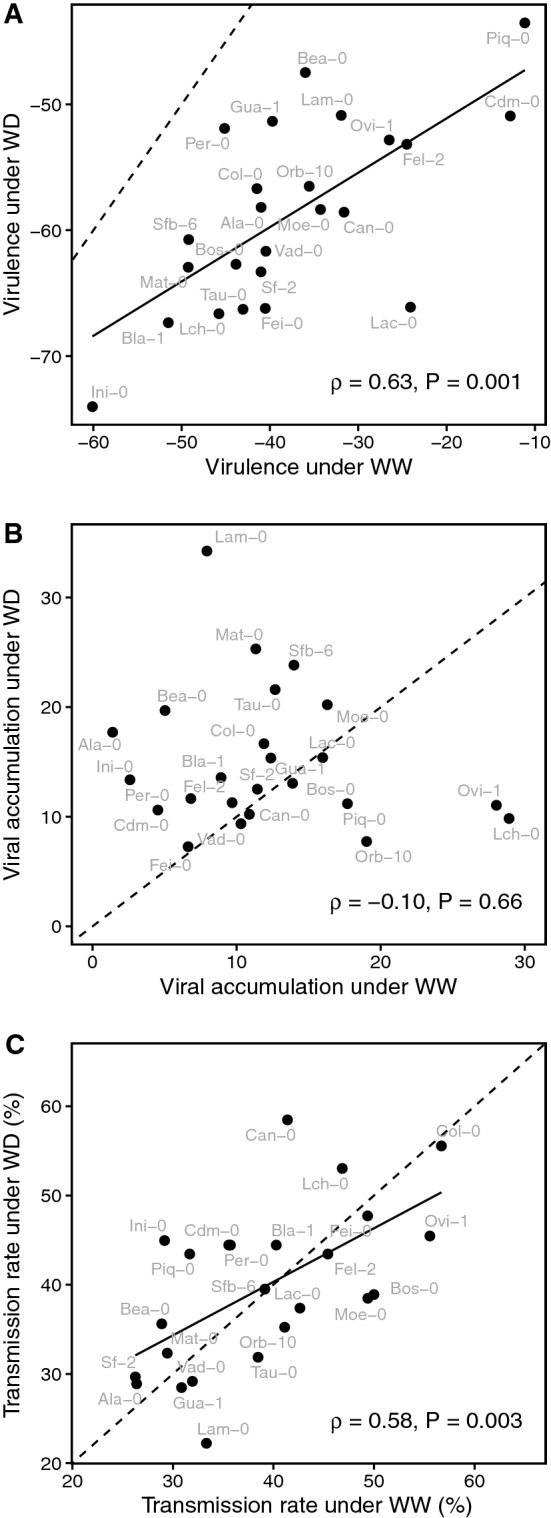
Effect of water deficit on CaMV traits in *A. thaliana* accessions. Relationships between mean values of (**A**) CaMV virulence (expressed as the % of vegetative growth reduction). (**B**) CaMV accumulation (N_0_ CaMV / N_0_ UBC21_Actin) or (**C**) transmission rate (%) under well-watered (WW) and water deficit (WD) treatments. Dashed lines represent 1:1 line, and solid lines represent significant linear regressions. Spearman’s correlation coefficients and associated *P *values are reported for each trait.

For instance, vegetative growth decreased from 43.5 ± 3.8% in Piq-0 to 74.0 ± 1.5% in Ini-0 (Fig. 2A). Bea-0 was the only accession that exhibited a similar reduction of vegetative growth under both watering conditions (Fig. 2A). Noticeably, variation of aboveground dry mass between accessions was significantly lower under WD than under WW (among-accession coefficient of variation = 31% under WW vs. 13% under WD; statistic of signed-likelihood ratio test = 107.8; *P* < 0.001). A positive and significant rank correlation between virulence under WW treatment and virulence under WD treatment was found (*⍴* = 0.63, *P* = 0.001; Fig. 3A). Thus, WD did not significantly affect the ranking of *A. thaliana* accessions according to CaMV virulence.

Viral accumulation estimated by qPCR varied greatly among accessions whatever the watering treatment (Fig. 2B, Supplementary Table S4). CaMV accumulation was 20-fold higher in Lch-0 than in Ala-0 accession when plants grew under WW conditions, while a fourfold CaMV accumulation difference was measured between Fei-0 and Lam-0 accessions in the WD treatment (Fig. 2B). Under WD, viral accumulation increased significantly, or marginally significantly, in seven accessions (Ini-0, Sfb-6, Ala-0, Bea-0, Lam-0, *P* < 0.05; Mat-0, Cdm-0, *P* < 0.10), while it decreased significantly in three others (Lch-0, Orb-10 and Ovi-1, *P* < 0.05) (Fig. 2B). Contrary to what was observed in virulence analyses, no correlation between viral accumulation under WW and under WD treatments was detected (*r* = –0.10, *P* = 0.66; Fig. 3B).

Under WW conditions, transmission rate varied from 26.3 ± 3.8% to 56.9 ± 4.2% (Sf-2 and Col-0, respectively) (Fig. 2C, Supplementary Table S5). Under WD, transmission rate varied also in a similar range (22.2 ± 5.2–58.5 ± 3.7% for Lam-0 and Can-0, respectively) (Fig. 2C). A significant and positive relationship between transmission rate under WW and WD treatments was detected (*r* = 0.58, *P* = 0.003; Fig. 3C). Thus, an accession for which CaMV transmission was high under WW showed also a high transmission efficiency under WD (Fig. 3C). Interestingly, a significant alteration of the transmission rate when source plants were subjected to WD was observed in four accessions. The transmission rate increased significantly in Can-0 (from 41 to 59%; *P* = 0.004) and Ini-0 (29% to 45%; *P* = 0.009), while it decreased significantly in Bos-0 (from 50 to 39%; *P* = 0.044) and Lam-0 (from 33 to 22%; *P* = 0.049) in response to the WD treatment (Fig. 2C).

### Water deficit changes relationships between viral traits

**Figure 4:**
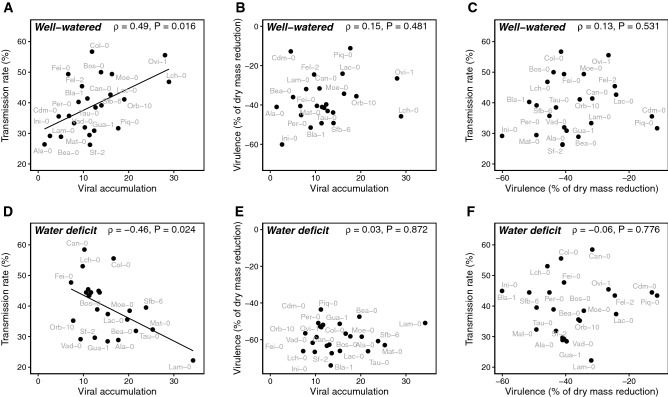
Relationships between CaMV traits in *A. thaliana* accessions under two contrasting watering regimes. Relationships between CaMV virulence on vegetative growth, transmission rate and accumulation under well-watered (**A**–**C**) and water deficit (**D**–**F**) conditions. Solid lines represent significant linear regressions. Spearman’s correlation coefficients and associated *P *values are reported for each relationship.

Under WW conditions, a significant and positive correlation was found between viral accumulation and CaMV transmission rate (*⍴* = 0.49, *P* = 0.016; Fig. 4A). Under this watering treatment, no significant rank correlation between viral accumulation and CaMV virulence could be detected (*⍴* = 0.15, *P* = 0.481; Fig. 4B). No significant correlation between CaMV virulence and viral transmission was also found under WW treatment (*⍴* = 0.13, *P* = 0.531; Fig. 4C). Changes in viral traits in response to WD across accessions (Fig. 2) led to an alteration of the relationships between these traits. Specifically, the correlation between viral accumulation and CaMV transmission was significantly reversed under WD (*⍴* = –0.46, *P* = 0.024; Fig. 4D). As observed in the WW treatment, no correlation between CaMV virulence and viral accumulation (*⍴* = 0.03, *P* = 0.872; Fig. 4E), or between virulence and viral transmission (*⍴* = − 0.06, *P* = 0.776; Fig. 4F), was observed.

### Relationships between viral traits and biogeographic origin of accessions

Correlations between climate at the collection sites of the accessions and viral traits were investigated (Supplementary Fig. S1). CaMV virulence was significantly positively correlated to isothermality—defined as the ratio of mean diurnal range of temperature (mean of monthly(max. temp—min. temp)) and temperature annual range—under WW, and positively, but not significantly, under WD (WW: *r* = 0.50, *P* = 0.04; WD: *r* = 0.27, *P* = 0.21; Supplementary Fig. S1). Viral accumulation under WW was significantly negatively correlated with precipitation seasonality, i.e. the coefficient of variation of monthly precipitations (WW: *r* = –0.43, *P* = 0.04; Supplementary Fig. S1). Virus accumulation under WD was significantly negatively correlated with isothermality (WD: *r* = –0.51, *P* = 0.014; Supplementary Fig. S1). Viral transmission was significantly correlated to isothermality only under WD conditions (WD: *r* = 0.42, *P* = 0.046; Fig. S1).

## Discussion

In a previous independent study, we showed that water deficit altered the transmission-virulence trade-off in the CaMV-*A. thaliana* pathosystem^10^. However, due to the restricted number of *A. thaliana* accessions, we were unable to test two key assumptions of the transmission-virulence trade-off hypothesis: (1) correlation between accumulation and transmission, and (2) correlation between accumulation and virulence. Here, we measured CaMV accumulation, virulence on vegetative growth and transmission rate by the aphid *M. persicae* in 24 natural Iberic accessions of *A. thaliana* presenting a large genetic variability and grown under two contrasting watering treatments^43^. To reflect variable plant responses levels to water deficit and, potentially, to virus infection, we selected the 24 natural accessions of *A. thaliana* from a large variety of climatic regions and altitudes distributed evenly across the Iberian Peninsula^44^. Significant relationships between climate parameters (isothermality, temperature and precipitation patterns and seasonality) and viral traits were detected depending of the watering treatment. Climatic conditions are frequently identified as factors of local adaptation of plant species. In *A. thaliana*, climatic factors have been found to be significantly associated to several traits, including flowering phenology, growth and functional strategies^45–47^, as well as genomic regions associated with regulation of gene expression, especially at regional and micro-geographic scales^48,49^. Contrary to a study by Montes and colleagues^9^, where no relationship between Cucumber mosaic virus viral accumulation and climatic variables from the original local populations could be detected, CaMV viral accumulation under well-watered conditions was significantly negatively correlated with precipitation seasonality while under WD, a negative correlation was detected between viral accumulation and isothermality. Interestingly, vectored-transmission was positively correlated to isothermality when plants were submitted to a WD. Under WD, CaMV accumulation within low temperature fluctuations environment-accessions was reduced while vectored-transmission would be higher than in other accessions. These relationships may result from the interrelationships between local climate conditions, occurrence of selective pathogens and the functional strategies of the plant genotypes^9,20^. The adaptive value of these relationships will require further investigations combining experiments under field and controlled conditions.

Plant signaling pathways and responses to various abiotic stresses partly overlap those induced by viral infection, and their mutual interference is not a novel concept. Indeed, the effect of abiotic plant stresses on viral accumulation and virulence through the hijacking of plant signaling and defense pathways has received recent attention^21,22,50^. In accordance with previous results^20^, we showed here that application of a water deficit to CaMV-infected *A. thaliana* leads to higher virulence, and was overall more detrimental to plant performance compared with virulence under well-watered condition regardless of accession. Under WD, CaMV accumulation was altered in 10 accessions; in most cases there was a significant increase of this viral trait. While a negative (or no) effect of WD on viral accumulation has already been shown in several viral pathosystems^10,25,31,32^, to our knowledge, such a positive effect on virus accumulation has not previously been reported.

In the context of climate change, a number of studies have investigated vectored-transmission in contrasted abiotic environments [for review see^51^]. WD applied to CaMV-source plants did not significantly impact CaMV transmission rate, except in four accessions. In these four accessions, WD had a contrasted effect on the transmission success, linked to the accession origin. While CaMV transmission by the aphid *M. persicae* increased in Can-0 and Ini-0 accessions under water deficit, it decreased in Bos-0 and Lam-0 accessions.

A positive correlation between CaMV transmission rate and accumulation was observed, which had not been detected when using fewer accessions^10^. This discrepancy might be explained by the different growing conditions (8-h vs. 12-h day length) used in the two studies. The positive correlation between CaMV transmission rate and accumulation observed here, and already shown in other pathosystems, might reflect the fact that a high availability of virus particles within plant cells increases the chance of acquisition by the vector^37,52^. However, in this study, factors other than accumulation might also explain alteration of virus transmission when plants experienced a water deficit. Indeed, in the specific case of Can-0 accession, which exhibited a significant increase of CaMV transmission under WD, accumulation did not seem to be the limiting factor as virus accumulation remained stable whatever the watering treatment. This observation was supported by the inverse relationship between CaMV transmission and virus accumulation under WD. As a result, plants with a lower virus content became significantly better source plants for vectored-transmission under WD. A lack of positive correlation between accumulation and transmission rate has already been shown in CaMV-infected *B. rapa* source plants experiencing severe WD^31^. The significant increase in transmission rate was suggested to be due to a change in host plant physiological status that could trigger a direct effect on virus behavior^53,54^. As a consequence, these rapid changes in virus behavior may actually predisposes the infected plant to a more efficient virus acquisition and transmission by aphid vectors^53,54^. This remarkable phenomenon has been termed ‘transmission activation’ and can be triggered by abiotic stresses such as CO_2_ treatment^55^.

We were unable to find any other relationship between viral traits. Unsuccessful validation of the trade-off hypothesis—i.e. a negative correlation between virulence and transmission—might be explained by the fact that one of the two required assumptions of this trade-off—i.e. a positive correlation between accumulation and virulence—was not demonstrated in our model system. Moreover, the trade-off hypothesis is an adaptive hypothesis that supposes a common evolutionary history between the host and the pathogen, suggesting efficient development of the parasite without harming the host^35^. Indeed, with the rise of metagenomics, sequence data collected *in natura* confirm that most virus-infected plants are asymptomatic^56^. Also, it is important to note that, in our system, the virus isolate did not co-evolve with these specific plant accessions even though several other CaMV isolates are reported to infect natural *A. thaliana* populations in Spain^41^. The isolate CaMV Cabb B-JI was originally isolated from *B. rapa* and fixed by cloning the genome sequence^57^. Moreover, the trade-off hypothesis, which assumes a simplified biology where virulence and transmission of the parasite are independent of the characteristics of the host, remains controversial^58^. In fact, viral traits are the result of multiple interactions within the host, such as immune responses to counteract the development of infection.

In conclusion, our results reaffirm that water deficit might have substantial effects on key viral traits, with epidemiological consequences for plant viral disease. The multi-faceted relationships between virulence, viral accumulation and vectored-transmission according to the environmental conditions experienced by the host invite further investigation.

## Methods

### Experimental design and *A. thaliana* growth treatments

We selected 24 natural accessions of *A. thaliana* originating from the Iberian Peninsula sequenced by Carlos Alonso-Blanco and collaborators and belonging to four distinct genetic lineages as determined by the 1001 genomes Project (S2 Fig, Supplementary Table S6) (http://1001genomes.org/))^44^. These accessions were grown in two independent experiments (experiment 1, *n* = 13; experiment 2, *n* = 12; Col-0 accession in common), under combinations of well-watered (WW), water deficit (WD), CaMV-inoculation (CaMV) and mock inoculation (Mock) treatments. Plants were analyzed in four treatments: Mock:WW (*n* = 4 plants per accession), Mock:WD (*n* = 4), CaMV:WW (*n* = 15) and CaMV:WD (*n* = 15) in each independent experiment.

Experiments were conducted in the PHENOPSIS facility. This phenotyping platform allows automated watering, weighing and imaging of 504 potted plants under strictly controlled environmental conditions^42^. Three to five seeds were sown at the soil surface in 225-ml pots filled with a 30:70 (v/v) mixture of clay and organic compost (substrate SP 15% KLASMANN) and placed randomly in the PHENOPSIS growth chamber. Soil water content was estimated for each pot before sowing, as previously described^42^. The soil surface was moistened with deionized water, and pots were placed in the dark for 2 days at 12 °C air temperature and 70% air relative humidity. Pots were dampened with sprayed deionized water three times a day until germination. After the germination phase (*ca.* 7 days), plants were cultivated under 12-h day length at 200 μmol m^–2^ s^–1^ photosynthetic photon flux density at plant height. Air temperature was set to 20 °C, and air relative humidity was adjusted in order to maintain constant water vapor pressure deficit at 0.6 kPa. At the appearance of the cotyledons, one plant was kept per pot, and the temperature was set at 21/18 °C day/night, while the vapor pressure deficit was set at 0.75 kPa. Each pot was weighed daily and watered with deionized water to reach the target soil relative water content. Soil relative water content was maintained at 1.4 g H_2_O g^–1^ dry soil (WW) until application of the treatments. CaMV- or mock-inoculation (see below) was performed at the emergence of the tenth rosette leaf. WD was applied 1 week after inoculation—the approximate timing of first symptom appearance. Irrigation of half of the CaMV- and mock-inoculated plants was stopped to reach WD treatment at 0.50 H_2_O g^–1^ dry soil, reached after 7 days of water deprivation, and then maintained at this value until the end of the experiment. Under WW, soil relative water content was maintained at 1.4 g H_2_O g^–1^ dry soil. All environmental data, including daily soil water content, air temperature, and vapor pressure deficit, are available in the PHENOPSIS database^59^.

### Virus purification and mechanical inoculation of source plants

The CaMV isolate Cabb B-JI—a non-circulative aphid-transmitted virus—was used in this study^57^. Virus particles were purified from CaMV-infected *Brassica rapa* cv. “Just Right” (turnip) plants as previously described^60^. The quality and quantity of purified virus were assessed by polyacrylamide gel electrophoresis under denaturing treatments (12% SDS-PAGE) and by spectrometric measurements at 230, 260, and 280 nm (NanoDrop 2000 spectrophotometer). Virus concentration was estimated by spectrometry using the formula described by Hull and Shepherd^60^. At the 10-leaf stage, *A. thaliana* source plants were mechanically inoculated as previously described^10^. Briefly, CaMV-infected turnip extract was prepared from 1 g of infected leaf material [turnip leaves presenting systemic symptoms collected at 21 days post inoculation (dpi)] ground in 1 mL of distilled water with carborundum. Purified CaMV particles were then added to this mix at a final concentration of 0.2 mg mL^–1^ to optimize infection success. For each inoculated plant, 10 μL of the solution described above was deposited on each of three middle-rank leaves. Leaves were then rubbed with an abrasive pestle. The control group was mock-inoculated in a similar way to mimic the wounds induced by mechanical inoculation. Mock-inoculation was performed with a mix containing non-infected turnip plant extract and the buffer used for virus purification (100 mM Tris–HCl, 2.5 mM MgCl_2_, pH 7). All plants were randomly inoculated, independently of accession and watering regime.

### Measurement of plant traits

Harvests were carried out at 30 dpi following transmission experiments (see below). Each rosette was cut, fresh mass was measured then the tissue was kept in deionized water for 24 h at 4 °C to determine the water-saturated weight (mg). Collected rosettes were subsequently oven-dried at 65 °C for at least 5 days, and their dry masses determined.

Virulence, described as the impact of CaMV infection on vegetative growth^61^, was estimated for each accession as the relative change of mean aboveground dry mass (ADM) of the rosette as: (ADM_CaMV:WW_ – ADM_Mock:WW_)*100 /ADM_Mock:WW_) and (ADM_CaMV:WD_ – ADM_Mock:WW_)*100 /ADM_Mock:WW_).

### Aphid rearing

The colony of the aphid-vector species *Myzus persicae*, collected over 30 years ago in the south of France, was maintained on eggplants (*Solanum melongena*) in insect-proof cages, in a growth chamber at 24/19 °C with a photoperiod of 14/10 h (day/night), ensuring clonal reproduction (G. Labonne, pers.comm.). Aphids were transferred to new cages and to new non-infested host plants (*S. melongena*) every 2 weeks, in order to avoid overcrowding and induction of the development of winged morphs.

### Transmission assays

CaMV transmission efficiency was assessed at 30 dpi. Batches of 20 M*. persicae* larvae (L2–L4 instars) were starved for 1 h before being transferred to the rosette center of a CaMV-mechanically infected source plant for virus acquisition; 11 symptomatic mechanically infected source plants were used per accession and watering treatment. When aphids stopped walking and inserted their stylets into the leaf surface, they were allowed to feed for a short 2-min period. Viruliferous aphids were then immediately collected in a Petri dish and individually transferred to 1-month-old Col-0 plantlets (receptor plants) grown under non-stressing treatments (one aphid per receptor plant; nine receptor plants per source plant) as described^10^. After an inoculation period of 3 h, aphids were eliminated by insecticide spray (0.2% Pirimor G). Receptor plants were then placed in a growth chamber with the same treatments of air humidity, temperature and light as source plants and maintained under non-stressing conditions. Symptoms of virus infection were recorded 21 days later by visual inspection on receptor plants, following the procedure previously reported^10^ and virus transmission rate was then calculated. Following transmission experiments, three leaf discs in the center of the rosette were randomly collected on each mechanically infected source plant and stored at – 80 °C for further nucleic acid extraction and virus quantification.

### Plant DNA extraction

Total DNA from CaMV-infected samples (pool of three leaf discs collected per plant) was extracted according to a modified Edwards’ protocol with an additional washing step with 70% ethanol^62^. DNA was resuspended in 50 μL of distilled water, and ten-fold dilutions were used as qPCR templates. Quality and quantity of the extracted total nucleic acid were assessed by spectroscopic measurements at 230, 260 and 280 nm (NanoDrop 2000 spectrophotometer).

### Viral accumulation

CaMV DNA quantification (11 biological replicates per accession and treatment) was performed in duplicate by qPCR in 384-well optical plates using the LightCycler FastStart DNA Master Plus SYBRGreen I kit (Roche) in a LightCycler 480 (Roche) thermocycler according to the manufacturer’s instructions. Specific primers designed for quantification of the CaMV genome (Ca4443-F: 5′-GACCTAAAAGTCATCAAGCCCA-3′ and Ca4557-R: 5′-TAGCTTTGTAGTTGACTACCATACG-3′) and two housekeeping genes: *A. thaliana* ubiquitin-conjugating enzyme 21 gene (*UBC21*; GenBank accession DQ027035; UBC21_At_F: 5′-TGCAACCTCCTCAAGTTCGA-3′ and UBC21_At_R: 5′-GCAGGACTCCAAGCATTCTT-3′) and *A. thaliana Actin* gene (GenBank accession GQ339782.1; F-Act2: 5′-GACYBTAYGGTAACATTGTGCTC-3′ and R-ActBra: 5′-GATCTCTTTGCTCATACGGTCTG-3′) were used at a final concentration of 0.3  μM. All qPCR reactions were performed with 40 cycles (95 °C for 15 s, 62 °C for 15 s and 72 °C for 15 s) after an initial step at 95 °C for 10 min. The qPCR data were analyzed with the LinReg PCR program to account for the efficiency of every single PCR reaction^63^. The absolute initial viral concentration in *A. thaliana* plants, expressed in arbitrary fluorescence units (N_0_ CaMV) was divided by that of *A. thaliana UBC21* and *Actin* genes, in order to normalize the amount of plant material analyzed in all samples.

### Data analyses

All analyzes were performed in the programming environment R^64^. Variations of vegetative growth (aboveground dry mass), viral accumulation and transmission rate among accessions in response to virus infection and watering treatment were analyzed in both parametric (type III) and non-parametric (rank-based) ANOVAs. Non-parametric procedure was used because of unbalanced sampling across factor levels and risks of deviation from Normality and heteroscedasticity. Data of the two experiments were analyzed together since no significant interactive or main effect of experiment was found for aboveground dry mass, viral accumulation, and viral transmission of the control accession Col-0 (Supplementary Table S7). For each accession, the effects of the treatments on traits were analysed by non-parametric tests for two (Wilcoxon) or more (Kruskal–Wallis) samples. For each accession, relative change was calculated as the ratio of the difference between the trait value of each replicate plant and mean trait value under control conditions (mock:well-watered) to the mean trait value under control conditions. Non-parametric ANOVAs were performed using the *raov* function of the Rfit package^65^. Bootstrapped 95% confidence intervals (CI) of mean trait values were computed following the *mean_cl_boot* procedure of the Hmisc package^66^. We used the R package cvequality (Version 0.1.3)^67^ to test for significant differences between coefficients of variation. Nonlinear models were fitted using the *nls* function and 95% confidence intervals for the parameters of fitted models were computed with the *confint* function of the package mass^68^. The effect of watering on transmission rate was analyzed using generalized linear models with the binomial model in the *glm* function of the stat package applied to the proportion of infected and uninfected plants in the transmission assays. Relationships between traits were examined with Spearman’s rank-order coefficients of correlation (*⍴*) using the function *cor.test*. Relationships between traits and climate at the collection sites of the accessions (obtained from WorldClim v. 1.4, http://worldclim.org)^69^ were examined in generalised least squares models in order to take spatial autocorrelation into account. First, we tested the bivariate relationships between traits and climatic variables, then we added a Gaussian autocorrelation structure to the model. Pearson’s correlation coefficients are reported with GLS *P *values.

### Statement on experimental research on plants

Seeds used in the present study were obtained with permission from Rebecca Schwab and Detlef Weigel (Max Planck Institute for Developmental Biology, Tuebingen, Germany) and comply with relevant institutional, national, and international guidelines and legislation.

## Supplementary Information

Supplementary Information.
