# Supplementary material for: Water deficit changes the relationships between epidemiological traits of Cauliflower mosaic virus across diverse Arabidopsis thaliana accessions
Source: Sci Rep. 2021 Dec 16;11:24103. doi: 10.1038/s41598-021-03462-x (PMC8677750; doi:10.1038/s41598-021-03462-x)
Supplement: Supplementary file 1 — Supplementary Information. [file 41598_2021_3462_MOESM1_ESM.docx]

**Supporting information**


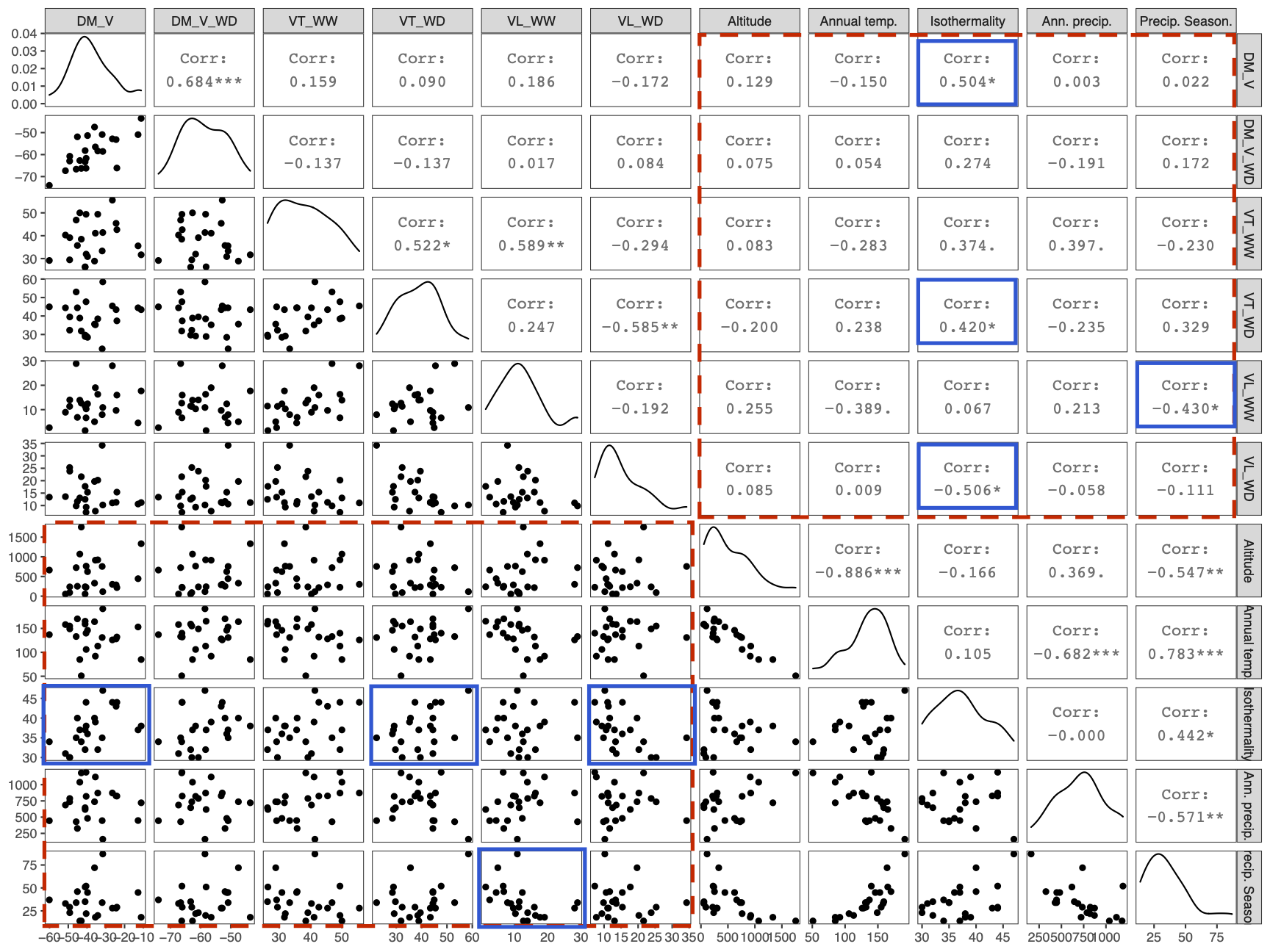


**Figure S1. Relationships between traits of the accessions measured in this study and altitude and climatic indicators at the collection sites of the accessions.** DM_V: aboveground dry mass change in response to viral infection under well-watered (WW) conditions; DM_V_WD: aboveground dry mass change in response to viral infection under water deficit (WD) conditions; VT_WW: viral transmission under WW conditions; VT_WD: viral transmission under WD conditions; VL_WW: viral accumulation under WW conditions; VT_WW: viral accumulation under WD conditions. Annual temp: annual mean temperature (BIO1); Ann. Precip: annual mean precipitations (BIO3); Precip Season. (BIO12); precipitation seasonality (BIO15). Corr.: Pearson’s coefficient of correlation. Dashed red delimited zone corresponds to the relationships between traits and bioclimatic variables. Significant (*: P < 0.05; **: P < 0.01; ***: P < 0.001) correlation coefficients, after correction for spatial autocorrelation using generalized mean squares models, are indicated by blue squares.

**Figure S2. Location and climatic conditions of the accessions collecting sites.** Distribution of the 24 iberian natural accessions used in this study. Color of the points represents the altitude at each accession’s collection point (http://1001genomes.org/). The map was generated using R (v.4.0) maps package v.3.4 (<https://CRAN.R-project.org/package=maps>) and shaded following the temperature gradient as obtained from WorldClim climate data^69^.

**Table S1.** Mean, 95% CI, minimum and maximum value of aboveground dry mass (mg) at 30 dpi of *Arabidopsis thaliana* accessions mock-inoculated (Mock) or inoculated by the *Cauliflower mosaic virus* (CaMV) grown under well-watered (WW) and water deficit (WD) treatments.

| **Accession** | **watering** | **inoculation** | **mean_AbovegroundDryMass** | **lwr_95ci** | **upr_95ci** | **min** | **max** |
| --- | --- | --- | --- | --- | --- | --- | --- |
| Bla-1 | WW | Mock | 928.5900 | 737.3719 | 1119.8081 | 811.50 | 1096.24 |
| Bla-1 | WW | CaMV | 450.2036 | 350.4015 | 550.0058 | 254.02 | 836.80 |
| Bla-1 | WD | Mock | 480.4625 | 373.9688 | 586.9562 | 422.00 | 575.10 |
| Bla-1 | WD | CaMV | 303.2627 | 256.9516 | 349.5737 | 166.02 | 491.30 |
| Can-0 | WW | Mock | 784.6189 | 744.8305 | 824.4073 | 684.00 | 867.60 |
| Can-0 | WW | CaMV | 543.7485 | 480.9946 | 606.5023 | 356.70 | 714.10 |
| Can-0 | WD | Mock | 524.2667 | 434.3000 | 614.2334 | 382.18 | 763.00 |
| Can-0 | WD | CaMV | 329.4064 | 276.0737 | 382.7392 | 182.24 | 529.12 |
| Col-0_E1 | WW | Mock | 736.7857 | 659.4562 | 814.1152 | 578.24 | 834.16 |
| Col-0_E1 | WW | CaMV | 372.6514 | 315.4475 | 429.8553 | 161.05 | 530.50 |
| Col-0_E1 | WD | Mock | 428.5933 | 300.2913 | 556.8953 | 275.80 | 566.14 |
| Col-0_E1 | WD | CaMV | 271.4827 | 233.9305 | 309.0348 | 183.00 | 377.18 |
| Fei-0 | WW | Mock | 724.9600 | 577.7728 | 872.1472 | 642.85 | 818.79 |
| Fei-0 | WW | CaMV | 431.1653 | 351.6784 | 510.6523 | 175.80 | 696.34 |
| Fei-0 | WD | Mock | 410.0750 | 305.2086 | 514.9414 | 325.20 | 485.60 |
| Fei-0 | WD | CaMV | 244.9750 | 208.2610 | 281.6890 | 145.00 | 348.48 |
| Bos-0 | WW | Mock | 852.6950 | 747.0568 | 958.3332 | 807.30 | 949.88 |
| Bos-0 | WW | CaMV | 478.8100 | 396.8461 | 560.7739 | 210.85 | 647.18 |
| Bos-0 | WD | Mock | 513.9875 | 444.5040 | 583.4710 | 467.10 | 570.25 |
| Bos-0 | WD | CaMV | 318.0121 | 254.5625 | 381.4618 | 181.50 | 525.80 |
| Fel-2 | WW | Mock | 809.1975 | 538.1121 | 1080.2829 | 634.30 | 1031.50 |
| Fel-2 | WW | CaMV | 610.9331 | 542.8421 | 679.0240 | 413.10 | 839.00 |
| Fel-2 | WD | Mock | 475.2750 | 239.1515 | 711.3985 | 360.00 | 671.80 |
| Fel-2 | WD | CaMV | 378.9340 | 335.0967 | 422.7713 | 183.09 | 492.22 |
| Ini-0 | WW | Mock | 927.0350 | 793.5430 | 1060.5270 | 855.10 | 1038.50 |
| Ini-0 | WW | CaMV | 369.8320 | 224.2699 | 515.3941 | 222.78 | 551.34 |
| Ini-0 | WD | Mock | 513.0450 | 336.9327 | 689.1573 | 360.34 | 610.00 |
| Ini-0 | WD | CaMV | 240.8850 | 210.6878 | 271.0822 | 170.78 | 323.90 |
| Lac-0 | WW | Mock | 670.9750 | 581.7775 | 760.1725 | 589.00 | 714.40 |
| Lac-0 | WW | CaMV | 509.3762 | 442.9305 | 575.8218 | 302.34 | 697.20 |
| Lac-0 | WD | Mock | 343.9650 | 197.9832 | 489.9468 | 235.50 | 424.44 |
| Lac-0 | WD | CaMV | 227.3500 | 188.8862 | 265.8138 | 104.76 | 365.30 |
| Lch-0 | WW | Mock | 771.0700 | 610.4076 | 931.7324 | 672.20 | 912.16 |
| Lch-0 | WW | CaMV | 418.0221 | 350.1114 | 485.9329 | 230.56 | 694.20 |
| Lch-0 | WD | Mock | 416.2675 | 349.5754 | 482.9596 | 374.00 | 471.97 |
| Lch-0 | WD | CaMV | 257.2880 | 217.9062 | 296.6698 | 161.75 | 368.36 |
| Mat-0 | WW | Mock | 1086.6000 | 929.9032 | 1243.2968 | 980.40 | 1216.10 |
| Mat-0 | WW | CaMV | 551.3255 | 490.5595 | 612.0915 | 397.54 | 721.00 |
| Mat-0 | WD | Mock | 516.0550 | 375.7642 | 656.3458 | 384.22 | 565.88 |
| Mat-0 | WD | CaMV | 402.6887 | 334.4107 | 470.9667 | 190.60 | 666.87 |
| Sfb-6 | WW | Mock | 883.5933 | 668.1032 | 1099.0834 | 801.20 | 974.12 |
| Sfb-6 | WW | CaMV | 448.7933 | 370.1969 | 527.3898 | 233.65 | 670.00 |
| Sfb-6 | WD | Mock | 548.2350 | 431.6450 | 664.8250 | 472.60 | 619.40 |
| Sfb-6 | WD | CaMV | 346.8053 | 311.1496 | 382.4611 | 219.10 | 435.00 |
| Tau-0 | WW | Mock | 964.2000 | 860.7658 | 1067.6342 | 877.00 | 1033.40 |
| Tau-0 | WW | CaMV | 549.0279 | 471.4065 | 626.6492 | 389.18 | 905.10 |
| Tau-0 | WD | Mock | 479.1500 | 387.2542 | 571.0458 | 413.40 | 547.40 |
| Tau-0 | WD | CaMV | 325.0950 | 260.3456 | 389.8444 | 190.50 | 650.78 |
| Sf-2 | WW | Mock | 999.5725 | 737.2762 | 1261.8688 | 768.30 | 1148.90 |
| Sf-2 | WW | CaMV | 589.5157 | 501.4858 | 677.5457 | 326.40 | 900.50 |
| Sf-2 | WD | Mock | 477.9525 | 295.2977 | 660.6073 | 308.40 | 558.08 |
| Sf-2 | WD | CaMV | 366.7293 | 320.7457 | 412.7129 | 223.90 | 522.50 |
| Cdm-0 | WW | Mock | 869.7100 | 679.8356 | 1059.5844 | 716.00 | 999.07 |
| Cdm-0 | WW | CaMV | 758.5425 | 530.9417 | 986.1433 | 461.80 | 1180.10 |
| Cdm-0 | WD | Mock | 650.8600 | 455.6525 | 846.0675 | 566.00 | 830.99 |
| Cdm-0 | WD | CaMV | 426.8100 | 373.9182 | 479.7018 | 276.40 | 573.60 |
| Col-0_E2 | WW | Mock | 609.5750 | 492.4359 | 726.7141 | 445.60 | 777.00 |
| Col-0_E2 | WW | CaMV | 405.1286 | 230.2450 | 580.0121 | 106.40 | 713.10 |
| Col-0_E2 | WD | Mock | 511.4567 | 403.6524 | 619.2610 | 327.40 | 627.90 |
| Col-0_E2 | WD | CaMV | 303.3400 | 260.8800 | 345.8000 | 140.80 | 463.50 |
| Ala-0 | WW | Mock | 824.5300 | 471.2423 | 1177.8177 | 568.00 | 1105.80 |
| Ala-0 | WW | CaMV | 486.4444 | 302.6412 | 670.2477 | 198.60 | 943.70 |
| Ala-0 | WD | Mock | 547.5450 | 369.9099 | 725.1801 | 429.09 | 675.00 |
| Ala-0 | WD | CaMV | 344.7814 | 277.3111 | 412.2517 | 208.60 | 604.10 |
| Bea-0 | WW | Mock | 830.2425 | 523.0496 | 1137.4354 | 661.44 | 1039.30 |
| Bea-0 | WW | CaMV | 531.3727 | 372.7579 | 689.9875 | 188.00 | 995.30 |
| Bea-0 | WD | Mock | 661.2550 | 599.9432 | 722.5668 | 604.00 | 684.28 |
| Bea-0 | WD | CaMV | 436.0909 | 309.0198 | 563.1620 | 135.90 | 696.90 |
| Gua-1 | WW | Mock | 978.7200 | 822.3906 | 1135.0494 | 922.86 | 1046.90 |
| Gua-1 | WW | CaMV | 589.9283 | 497.1697 | 682.6870 | 344.50 | 828.20 |
| Gua-1 | WD | Mock | 602.9000 | 426.7466 | 779.0534 | 482.00 | 701.20 |
| Gua-1 | WD | CaMV | 476.1786 | 390.4266 | 561.9305 | 282.60 | 658.56 |
| Lam-0 | WW | Mock | 617.0150 | 430.4429 | 803.5871 | 526.00 | 789.10 |
| Lam-0 | WW | CaMV | 420.0289 | 287.7815 | 552.2763 | 262.60 | 749.30 |
| Lam-0 | WD | Mock | 404.5950 | 176.0171 | 633.1729 | 200.20 | 527.20 |
| Lam-0 | WD | CaMV | 303.0513 | 267.6764 | 338.4262 | 214.60 | 421.17 |
| Moe-0 | WW | Mock | 826.7000 | 740.4938 | 912.9062 | 785.00 | 905.70 |
| Moe-0 | WW | CaMV | 543.3058 | 434.2692 | 652.3425 | 301.30 | 936.20 |
| Moe-0 | WD | Mock | 565.4800 | 311.2434 | 819.7166 | 401.02 | 711.70 |
| Moe-0 | WD | CaMV | 344.2714 | 276.2139 | 412.3289 | 176.70 | 630.30 |
| Orb-10 | WW | Mock | 785.5700 | 700.8873 | 870.2527 | 706.98 | 819.20 |
| Orb-10 | WW | CaMV | 506.2400 | 442.5402 | 569.9398 | 230.00 | 680.40 |
| Orb-10 | WD | Mock | 476.4500 | 346.9052 | 605.9948 | 386.30 | 555.90 |
| Orb-10 | WD | CaMV | 341.6385 | 265.6271 | 417.6498 | 178.40 | 590.50 |
| Ovi-1 | WW | Mock | 833.9000 | 590.2876 | 1077.5124 | 627.90 | 992.40 |
| Ovi-1 | WW | CaMV | 612.9809 | 520.7103 | 705.2515 | 425.30 | 830.60 |
| Ovi-1 | WD | Mock | 564.6725 | 300.6548 | 828.6902 | 341.76 | 697.43 |
| Ovi-1 | WD | CaMV | 393.3400 | 351.3947 | 435.2853 | 278.70 | 617.50 |
| Per-0 | WW | Mock | 500.2150 | 370.8418 | 629.5882 | 418.26 | 590.70 |
| Per-0 | WW | CaMV | 274.4091 | 249.0675 | 299.7507 | 210.70 | 353.70 |
| Per-0 | WD | Mock | 336.9600 | 108.5397 | 565.3803 | 164.10 | 507.00 |
| Per-0 | WD | CaMV | 240.5400 | 209.9214 | 271.1586 | 143.40 | 345.00 |
| Piq-0 | WW | Mock | 601.0000 | 302.4855 | 899.5145 | 389.30 | 818.00 |
| Piq-0 | WW | CaMV | 534.0692 | 419.8728 | 648.2656 | 167.40 | 818.00 |
| Piq-0 | WD | Mock | 474.0400 | 347.6827 | 600.3973 | 368.16 | 536.00 |
| Piq-0 | WD | CaMV | 339.3215 | 289.4721 | 389.1709 | 192.88 | 466.30 |
| Vad-0 | WW | Mock | 797.7750 | 536.7081 | 1058.8419 | 592.00 | 930.60 |
| Vad-0 | WW | CaMV | 474.9636 | 387.1007 | 562.8266 | 283.80 | 690.90 |
| Vad-0 | WD | Mock | 476.9900 | 374.9062 | 579.0738 | 430.40 | 571.90 |
| Vad-0 | WD | CaMV | 305.7829 | 247.7301 | 363.8357 | 156.90 | 470.80 |

**Table S2.** Mean, 95% CI, minimum and maximum value of percent change in aboveground dry mass (mg) at 30 dpi of *Arabidopsis thaliana* accessions in response to infection by *Cauliflower mosaic virus* (CaMV) and grown under well-watered (WW) and water deficit (WD) treatments.

| **Accession** | **watering** | **mean_prct_change_DM** | **lwr_se** | **upr_se** | **min** | **max** |
| --- | --- | --- | --- | --- | --- | --- |
| Ini-0 | WW | -60.10593 | -65.76133 | -54.450531 | -75.96854 | -40.5265173 |
| Ini-0 | WD | -74.01554 | -75.52334 | -72.507750 | -81.57783 | -65.0606504 |
| Bla-1 | WW | -51.51750 | -56.34113 | -46.693874 | -72.64455 | -9.8848792 |
| Bla-1 | WD | -67.34160 | -69.66688 | -65.016310 | -82.12128 | -47.0918274 |
| Mat-0 | WW | -49.26142 | -51.77127 | -46.751561 | -63.41432 | -33.6462360 |
| Mat-0 | WD | -62.94049 | -65.87021 | -60.010761 | -82.45905 | -38.6278299 |
| Sfb-6 | WW | -49.20816 | -53.24957 | -45.166745 | -73.55684 | -24.1732622 |
| Sfb-6 | WD | -60.75057 | -62.63202 | -58.869119 | -75.20352 | -50.7692076 |
| Lch-0 | WW | -45.78675 | -49.86352 | -41.709967 | -70.09869 | -9.9692635 |
| Lch-0 | WD | -66.63234 | -69.01366 | -64.251023 | -79.02266 | -52.2274242 |
| Per-0 | WW | -45.14177 | -47.41548 | -42.868058 | -57.87811 | -29.2904051 |
| Per-0 | WD | -51.91268 | -54.76661 | -49.058741 | -71.33233 | -31.0296572 |
| Col-0 | WW | -44.12773 | -48.83375 | -39.421707 | -82.54522 | 16.9831440 |
| Col-0 | WD | -56.69528 | -59.00764 | -54.382923 | -76.90194 | -23.9634171 |
| Bos-0 | WW | -43.84745 | -48.21474 | -39.480161 | -75.27252 | -24.1018184 |
| Bos-0 | WD | -62.70505 | -66.14941 | -59.260702 | -78.71455 | -38.3366855 |
| Tau-0 | WW | -43.05872 | -46.78509 | -39.332346 | -59.63700 | -6.1294337 |
| Tau-0 | WD | -66.28345 | -69.39187 | -63.175020 | -80.24269 | -32.5057042 |
| Sf-2 | WW | -41.02322 | -45.09972 | -36.946709 | -67.34604 | -9.9114872 |
| Sf-2 | WD | -63.31138 | -65.45627 | -61.166495 | -77.60042 | -47.7276536 |
| Ala-0 | WW | -41.00343 | -50.67032 | -31.336538 | -75.91355 | 14.4530824 |
| Ala-0 | WD | -58.18449 | -61.97221 | -54.396765 | -74.70074 | -26.7340182 |
| Fei-0 | WW | -40.52564 | -45.63772 | -35.413558 | -75.75039 | -3.9478040 |
| Fei-0 | WD | -66.20848 | -68.55265 | -63.864307 | -79.99890 | -51.9311410 |
| Vad-0 | WW | -40.46396 | -45.40688 | -35.521047 | -64.42606 | -13.3966344 |
| Vad-0 | WD | -61.67054 | -65.03887 | -58.302207 | -80.33280 | -40.9858669 |
| Gua-1 | WW | -39.72450 | -44.03055 | -35.418457 | -64.80096 | -15.3792709 |
| Gua-1 | WD | -51.34680 | -55.40243 | -47.291179 | -71.12555 | -32.7121138 |
| Bea-0 | WW | -35.99789 | -44.57215 | -27.423632 | -77.35601 | 19.8806373 |
| Bea-0 | WD | -47.47427 | -54.34337 | -40.605178 | -83.63129 | -16.0606690 |
| Orb-10 | WW | -35.55762 | -39.33829 | -31.776947 | -70.72190 | -13.3877312 |
| Orb-10 | WD | -56.51076 | -60.95168 | -52.069829 | -77.29038 | -24.8316509 |
| Moe-0 | WW | -34.28017 | -40.27266 | -28.287680 | -63.55389 | 13.2454337 |
| Moe-0 | WD | -58.35594 | -62.16660 | -54.545282 | -78.62586 | -23.7571066 |
| Lam-0 | WW | -31.92566 | -41.22027 | -22.631048 | -57.44026 | 21.4395112 |
| Lam-0 | WD | -50.88428 | -53.55739 | -48.211183 | -65.21965 | -31.7407194 |
| Can-0 | WW | -31.60497 | -35.22779 | -27.982151 | -55.13273 | -10.1774188 |
| Can-0 | WD | -58.56584 | -61.67106 | -55.460617 | -77.07707 | -33.4450019 |
| Ovi-1 | WW | -26.49228 | -31.45828 | -21.526274 | -48.99868 | -0.3957309 |
| Ovi-1 | WD | -52.83127 | -55.17651 | -50.486044 | -66.57873 | -25.9503538 |
| Fel-2 | WW | -24.50136 | -28.36339 | -20.639339 | -48.94942 | 3.6829699 |
| Fel-2 | WD | -53.17163 | -55.69747 | -50.645792 | -77.37388 | -39.1718338 |
| Lac-0 | WW | -24.08418 | -28.62925 | -19.539116 | -54.94020 | 3.9084914 |
| Lac-0 | WD | -66.11647 | -68.78925 | -63.443699 | -84.38690 | -45.5568389 |
| Cdm-0 | WW | -12.78213 | -23.84932 | -1.714948 | -46.90184 | 35.6889078 |
| Cdm-0 | WD | -50.92502 | -53.74007 | -48.109971 | -68.21929 | -34.0469812 |
| Piq-0 | WW | -11.13657 | -19.85740 | -2.415733 | -72.14642 | 36.1064892 |
| Piq-0 | WD | -43.54051 | -47.34736 | -39.733661 | -67.90682 | -22.4126456 |

**Table S3.** Results of ANOVA and robust (rank based) ANOVA for aboveground dry mass (log_10_; *n* = 6-14), within-host viral accumulation (*n* = 8-19) and viral transmission (*n* = 9-11) for all *A. thaliana* accessions grown under well-watered and water deficit conditions and mock- or CaMV-inoculated.

|  |  |  | | **ANOVA** | |  | **Robust ANOVA** | |
| --- | --- | --- | --- | --- | --- | --- | --- | --- |
| **Trait** | **Effect** | **df** | **F** | | **P-value** |  | **F** | **P-value** |
| Abvgrd dry mass | Accession | 24 | 14.207 | | **< 0.001** |  | 9.271 | **< 0.001** |
|  | Inoculation | 1 | 480.47 | | **< 0.001** |  | 474.6 | **< 0.001** |
|  | Watering | 1 | 614.38 | | **< 0.001** |  | 449.2 | **< 0.001** |
|  | Accession*Inoculation | 24 | 1.559 | | **0.043** |  | 1.773 | **0.013** |
|  | Accession*Watering | 24 | 2.210 | | **< 0.001** |  | 1.687 | **0.021** |
|  | Inoculation*Watering | 1 | 4.019 | | **0.045** |  | 3.544 | ***0.060*** |
|  | Acc*Inoc*Watering | 24 | 0.801 | | 0.738 |  | 0.873 | 0.640 |
|  |  |  |  | |  |  |  |  |
| Viral accumulation | Accession | 23 | 2.03 | | **0.004** |  | 3.202 | **< 0.001** |
|  | Watering | 1 | 8.87 | | **0.003** |  | 18.06 | **< 0.001** |
|  | Accession*Watering | 23 | 2.62 | | **< 0.001** |  | 3.801 | **< 0.001** |
|  |  |  |  | |  |  |  |  |
| Viral transmission | Accession | 23 | 5.926 | | **< 0.001** |  | 7.243 | **< 0.001** |
|  | Watering | 1 | 0.069 | | 0.792 |  | 0.854 | 0.356 |
|  | Accession*Watering | 23 | 1.198 | | 0.241 |  | 1.408 | ***0.099*** |

**Table S4.** Mean, 95% CI, minimum and maximum value of CaMV-accumulation in source plants (n = 11 per accession and per treatment) of *Arabidopsis thaliana* accessions grown under well-watered (WW) and water deficit (WD) treatments.

| **Accession** | **watering** | **mean_N0_CAMV__N0_ACTINE_UBC** | **lwr_se** | **upr_se** | **min** | **max** |
| --- | --- | --- | --- | --- | --- | --- |
| Ini-0 | WW | 2.595391 | 0.4563815 | 4.734400 | 0.3000000 | 11.146952 |
| Ini-0 | WD | 13.368478 | 8.3160179 | 18.420939 | 0.0747454 | 54.471148 |
| Bla-1 | WW | 8.920295 | 6.9306782 | 10.909912 | 0.3371296 | 18.341140 |
| Bla-1 | WD | 13.580931 | 11.1152288 | 16.046633 | 2.8535960 | 28.400000 |
| Mat-0 | WW | 11.334129 | 6.8699650 | 15.798294 | 0.3792558 | 40.500000 |
| Mat-0 | WD | 25.310106 | 18.4539537 | 32.166258 | 1.2400000 | 65.061526 |
| Sfb-6 | WW | 13.978031 | 11.4949141 | 16.461147 | 0.9371426 | 28.860000 |
| Sfb-6 | WD | 23.832114 | 18.7177612 | 28.946466 | 3.2100000 | 56.310000 |
| Lch-0 | WW | 28.907631 | 25.2457673 | 32.569494 | 18.4259338 | 55.000000 |
| Lch-0 | WD | 9.839396 | 7.0338776 | 12.644915 | 2.0171195 | 33.609478 |
| Per-0 | WW | 6.827097 | 4.2832266 | 9.370968 | 0.4413182 | 16.433554 |
| Per-0 | WD | 11.659676 | 9.5773555 | 13.741998 | 0.8296747 | 22.264379 |
| Col-0 | WW | 11.954541 | 9.8947338 | 14.014349 | 0.7275992 | 35.100000 |
| Col-0 | WD | 16.386171 | 14.1977006 | 18.574641 | 0.6634712 | 34.433594 |
| Bos-0 | WW | 13.885000 | 11.4261292 | 16.343871 | 0.2774257 | 25.312909 |
| Bos-0 | WD | 13.056097 | 11.3220048 | 14.790189 | 6.4803073 | 22.860000 |
| Tau-0 | WW | 12.672247 | 7.9605030 | 17.383992 | 0.8987879 | 43.452558 |
| Tau-0 | WD | 21.605116 | 17.1482396 | 26.061992 | 8.5000000 | 55.142593 |
| Sf-2 | WW | 11.446162 | 9.0883585 | 13.803965 | 1.1900000 | 25.720000 |
| Sf-2 | WD | 12.855397 | 10.1849147 | 15.525880 | 2.0984896 | 24.600000 |
| Ala-0 | WW | 1.397784 | 0.9281380 | 1.867429 | 0.2703911 | 3.072705 |
| Ala-0 | WD | 17.706961 | 11.8041666 | 23.609755 | 0.7431496 | 46.357555 |
| Fei-0 | WW | 6.639202 | 4.5970011 | 8.681403 | 0.3300000 | 24.000000 |
| Fei-0 | WD | 7.271807 | 5.9281846 | 8.615430 | 0.2121919 | 16.400000 |
| Vad-0 | WW | 10.305677 | 7.6455194 | 12.965835 | 0.6555448 | 23.656615 |
| Vad-0 | WD | 9.359979 | 7.7895090 | 10.930449 | 4.2702501 | 15.908820 |
| Gua-1 | WW | 12.374481 | 9.4256669 | 15.323294 | 4.2704928 | 22.560288 |
| Gua-1 | WD | 15.357002 | 10.8628550 | 19.851148 | 1.4337852 | 30.809497 |
| Bea-0 | WW | 5.035695 | 4.3503066 | 5.721083 | 3.1765406 | 7.239551 |
| Bea-0 | WD | 19.691212 | 12.6031328 | 26.779292 | 0.2478679 | 69.608120 |
| Orb-10 | WW | 19.022871 | 14.4299720 | 23.615769 | 2.2875930 | 39.745186 |
| Orb-10 | WD | 7.739431 | 6.3724757 | 9.106387 | 2.9565494 | 13.385595 |
| Moe-0 | WW | 16.299608 | 13.2174077 | 19.381809 | 3.5077294 | 34.265617 |
| Moe-0 | WD | 20.216393 | 15.2702908 | 25.162495 | 0.3211341 | 42.858182 |
| Lam-0 | WW | 7.937424 | 5.2717141 | 10.603134 | 0.2656894 | 19.803842 |
| Lam-0 | WD | 34.236277 | 24.2640951 | 44.208459 | 0.9989961 | 88.567638 |
| Can-0 | WW | 10.882189 | 8.6728952 | 13.091482 | 0.7500000 | 23.631603 |
| Can-0 | WD | 10.223564 | 8.0492159 | 12.397912 | 0.4011860 | 23.600000 |
| Ovi-1 | WW | 28.032391 | 22.7297213 | 33.335061 | 1.0119072 | 50.806759 |
| Ovi-1 | WD | 11.047631 | 8.9633067 | 13.131956 | 0.7925711 | 20.021992 |
| Fel-2 | WW | 9.689427 | 7.5298621 | 11.848992 | 1.7914152 | 19.918497 |
| Fel-2 | WD | 11.284438 | 9.1370595 | 13.431817 | 2.2400000 | 26.020000 |
| Lac-0 | WW | 15.973039 | 13.2839400 | 18.662138 | 6.9045276 | 27.100000 |
| Lac-0 | WD | 15.773683 | 9.8098416 | 21.737524 | 1.1777734 | 56.880000 |
| Cdm-0 | WW | 4.538695 | 2.4436639 | 6.633725 | 0.8461487 | 11.431090 |
| Cdm-0 | WD | 10.609660 | 7.7965670 | 13.422753 | 1.3768821 | 25.038076 |
| Piq-0 | WW | 17.690443 | 13.4825226 | 21.898363 | 0.3938768 | 38.791434 |
| Piq-0 | WD | 11.189611 | 7.9525287 | 14.426694 | 0.9413425 | 33.540009 |

**Table S5.** Mean, 95% CI, minimum and maximum value of transmission rate (i.e., mean proportion of infected receptor plants of *Arabidopsis thaliana* accessions infected by *Cauliflower mosaic virus* (CaMV) and grown under well-watered (WW) and water deficit (WD) treatments.

| **Accession** | **watering** | **mean_infectionRate** | **lwr_se** | **upr_se** | **min** | **max** |
| --- | --- | --- | --- | --- | --- | --- |
| Ini-0 | WW | 29.16667 | 23.71627 | 34.61706 | 12.50000 | 44.44444 |
| Ini-0 | WD | 44.94949 | 42.77133 | 47.12766 | 33.33333 | 55.55556 |
| Bla-1 | WW | 40.27778 | 34.82738 | 45.72817 | 11.11111 | 66.66667 |
| Bla-1 | WD | 44.44444 | 39.04253 | 49.84636 | 11.11111 | 66.66667 |
| Mat-0 | WW | 29.44444 | 23.60377 | 35.28512 | 0.00000 | 55.55556 |
| Mat-0 | WD | 32.32323 | 27.56295 | 37.08352 | 11.11111 | 55.55556 |
| Sfb-6 | WW | 39.14141 | 34.07861 | 44.20421 | 0.00000 | 62.50000 |
| Sfb-6 | WD | 39.50216 | 34.01196 | 44.99237 | 11.11111 | 62.50000 |
| Lch-0 | WW | 46.84343 | 45.04624 | 48.64063 | 37.50000 | 55.55556 |
| Lch-0 | WD | 53.03030 | 50.09405 | 55.96655 | 33.33333 | 66.66667 |
| Per-0 | WW | 35.71429 | 25.72773 | 45.70084 | 0.00000 | 66.66667 |
| Per-0 | WD | 44.44444 | 40.85183 | 48.03705 | 22.22222 | 55.55556 |
| Col-0 | WW | 56.94444 | 52.71970 | 61.16919 | 33.33333 | 88.88889 |
| Col-0 | WD | 55.55556 | 50.93194 | 60.17917 | 0.00000 | 88.88889 |
| Bos-0 | WW | 50.00000 | 46.64464 | 53.35536 | 33.33333 | 66.66667 |
| Bos-0 | WD | 38.88889 | 33.99094 | 43.78684 | 11.11111 | 66.66667 |
| Tau-0 | WW | 38.47222 | 33.13689 | 43.80756 | 11.11111 | 66.66667 |
| Tau-0 | WD | 31.87229 | 28.40993 | 35.33466 | 11.11111 | 50.00000 |
| Sf-2 | WW | 26.26263 | 22.51027 | 30.01498 | 0.00000 | 44.44444 |
| Sf-2 | WD | 29.65368 | 26.27278 | 33.03458 | 11.11111 | 44.44444 |
| Ala-0 | WW | 26.38889 | 18.00610 | 34.77168 | 0.00000 | 66.66667 |
| Ala-0 | WD | 28.88889 | 23.86494 | 33.91284 | 0.00000 | 44.44444 |
| Fei-0 | WW | 49.36869 | 45.63339 | 53.10399 | 25.00000 | 62.50000 |
| Fei-0 | WD | 47.70924 | 45.34952 | 50.06895 | 33.33333 | 55.55556 |
| Vad-0 | WW | 31.94444 | 25.84507 | 38.04382 | 0.00000 | 55.55556 |
| Vad-0 | WD | 29.16667 | 26.24387 | 32.08947 | 22.22222 | 44.44444 |
| Gua-1 | WW | 30.86420 | 23.74533 | 37.98306 | 11.11111 | 77.77778 |
| Gua-1 | WD | 28.47222 | 21.87892 | 35.06553 | 0.00000 | 50.00000 |
| Bea-0 | WW | 28.88889 | 22.01954 | 35.75824 | 0.00000 | 66.66667 |
| Bea-0 | WD | 35.60606 | 32.07612 | 39.13600 | 22.22222 | 55.55556 |
| Orb-10 | WW | 41.11111 | 36.12829 | 46.09393 | 22.22222 | 66.66667 |
| Orb-10 | WD | 35.22727 | 31.54655 | 38.90800 | 11.11111 | 55.55556 |
| Moe-0 | WW | 49.38272 | 42.93808 | 55.82735 | 22.22222 | 88.88889 |
| Moe-0 | WD | 38.47222 | 32.64532 | 44.29912 | 0.00000 | 66.66667 |
| Lam-0 | WW | 33.33333 | 30.90869 | 35.75798 | 22.22222 | 44.44444 |
| Lam-0 | WD | 22.22222 | 16.98439 | 27.46005 | 0.00000 | 44.44444 |
| Can-0 | WW | 41.38889 | 37.63460 | 45.14317 | 22.22222 | 55.55556 |
| Can-0 | WD | 58.47222 | 54.82420 | 62.12024 | 44.44444 | 77.77778 |
| Ovi-1 | WW | 55.55556 | 49.81780 | 61.29331 | 33.33333 | 88.88889 |
| Ovi-1 | WD | 45.45455 | 40.61027 | 50.29882 | 11.11111 | 66.66667 |
| Fel-2 | WW | 45.41667 | 40.79607 | 50.03726 | 22.22222 | 66.66667 |
| Fel-2 | WD | 43.43434 | 36.99817 | 49.87052 | 11.11111 | 77.77778 |
| Lac-0 | WW | 42.63889 | 36.68824 | 48.58954 | 12.50000 | 75.00000 |
| Lac-0 | WD | 37.37374 | 33.62138 | 41.12610 | 22.22222 | 66.66667 |
| Cdm-0 | WW | 35.55556 | 27.39059 | 43.72052 | 11.11111 | 55.55556 |
| Cdm-0 | WD | 44.44444 | 39.47540 | 49.41348 | 22.22222 | 66.66667 |
| Piq-0 | WW | 31.66667 | 24.54004 | 38.79329 | 0.00000 | 66.66667 |
| Piq-0 | WD | 43.43434 | 38.59007 | 48.27862 | 22.22222 | 66.66667 |

**Table S6.** Location and bioclimatic variables of *A. thaliana* accessions used in this study. Life cycle data were obtained from^70,71^.

| **idAccession** | **Life cycle** | **Long.** | **Lat.** | **Alt.** | **Mean Annual Temperature** | **Isothermality** | **Annual Precipitation** | **Precipitation Seasonality** |
| --- | --- | --- | --- | --- | --- | --- | --- | --- |
| **Bla-1** | Summer | 2.8 | 41.6833 | 54 | 158 | 31 | 686 | 33 |
| **Cdm-0** | Summer | -5.74 | 39.73 | 444 | 153 | 37 | 448 | 45 |
| **Fei-0** | Summer | -8.54 | 40.92 | 226 | 140 | 44 | 1191 | 52 |
| **Ala-0** | Summer | -6.89 | 39.72 | 239 | 165 | 37 | 618 | 51 |
| **Bea-0** | Summer | -5.27 | 36.52 | 330 | 164 | 40 | 737 | 72 |
| **Bos-0** | Summer | 0.69 | 42.78 | 1070 | 85 | 37 | 1038 | 14 |
| **Fel-2** | Summer | -5.7 | 43.31 | 287 | 128 | 43 | 827 | 28 |
| **Gua-1** | Summer | -5.33 | 39.4 | 623 | 146 | 36 | 481 | 45 |
| **Ini-0** | Summer | -3.75 | 40.46 | 663 | 137 | 34 | 445 | 37 |
| **Lac-0** | Summer | -5.91 | 43.33 | 218 | 132 | 44 | 824 | 29 |
| **Lam-0** | Summer | -3.89 | 40.57 | 755 | 131 | 35 | 446 | 34 |
| **Lch-0** | Summer | -4 | 40.51 | 725 | 133 | 35 | 429 | 34 |
| **Mat-0** | Summer | 2.69 | 41.76 | 91 | 155 | 30 | 735 | 29 |
| **Moe-0** | Summer | 2.37 | 41.78 | 923 | 113 | 32 | 871 | 20 |
| **Orb-10** | Summer | -1.23 | 42.97 | 914 | 92 | 39 | 1118 | 18 |
| **Ovi-1** | Summer | -5.87 | 43.38 | 301 | 126 | 44 | 872 | 28 |
| **Per-0** | Summer | -1.12 | 37.6 | 251 | 170 | 40 | 326 | 46 |
| **Piq-0** | Summer | -2.56 | 42.1 | 1333 | 85 | 38 | 721 | 18 |
| **Sfb-6** | Summer | 2.57 | 41.78 | 236 | 149 | 30 | 787 | 23 |
| **Tau-0** | Summer | 0.84 | 42.54 | 1756 | 51 | 34 | 1181 | 14 |
| **Vad-0** | Summer | -3.59 | 42.86 | 763 | 106 | 38 | 820 | 22 |
| **Sf-2** | Summer | 3.03333 | 41.7833 | 56 | 159 | 32 | 645 | 35 |

**Table S7.** Results of ANOVA and robust (rank based) ANOVA for aboveground dry mass (log_10_; *n* = 6-14), within-host viral accumulation (*n* = 8-19) and viral transmission (*n* = 9-11) for *A. thaliana* accession Col-0 grown under well-watered and water deficit conditions and mock- or CaMV-inoculated in two experiments.

|  |  |  | **ANOVA** | | **Robust ANOVA** | |
| --- | --- | --- | --- | --- | --- | --- |
| **Trait** | **Effect** | **df** | **F** | **P-value** | **F** | **P-value** |
| Abvgrd dry mass | Experiment | 1 | 0.202 | 0.654 | 0.416 | 0.521 |
|  | Inoculation | 1 | 25.25 | **< 0.001** | 25.43 | **< 0.001** |
|  | Watering | 1 | 53.88 | **< 0.001** | 57.30 | **< 0.001** |
|  | Experiment*Inoculation | 1 | 2.043 | 0.702 | 0.623 | 0.432 |
|  | Experiment*Watering | 1 | 0.148 | 0.157 | 2.45 | 0.122 |
|  | Inoculation*Watering | 1 | 0.837 | 0.363 | 0.072 | 0.789 |
|  | Experiment *Inoculation*Watering | 1 | 0.834 | 0.364 | 1.809 | 0.183 |
|  |  |  |  |  |  |  |
| Viral accumulation | Experiment | 1 | 0.097 | 0.757 | 0.08 | 0.772 |
|  | Watering | 1 | 2.757 | 0.103 | 2.479 | 0.121 |
|  | Experiment *Watering | 1 | 0.027 | 0.869 | 0.153 | 0.697 |
|  |  |  |  |  |  |  |
| Viral transmission | Experiment | 1 | 0.130 | 0.721 | 0.081 | 0.778 |
|  | Watering | 1 | 0.039 | 0.844 | 0.024 | 0.876 |
|  | Experiment *Watering | 1 | 0.136 | 0.714 | 0.142 | 0.708 |
